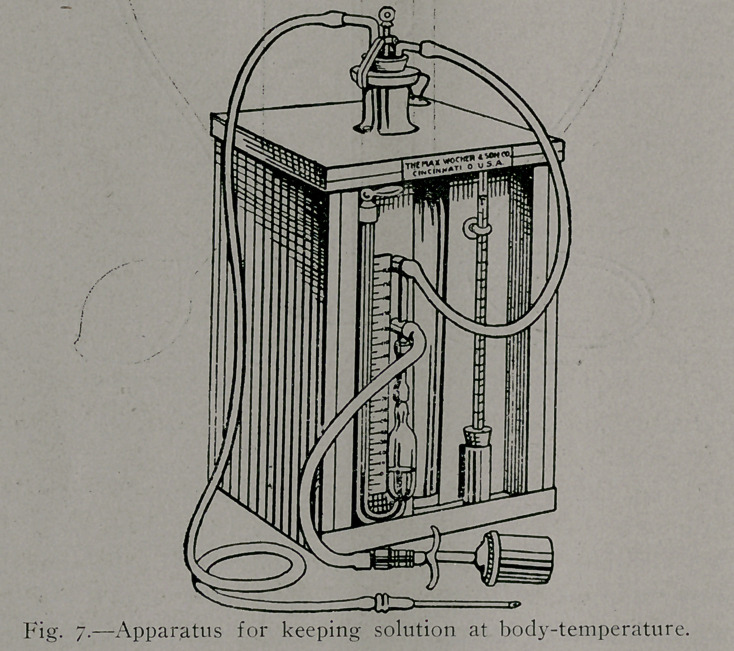# Selections and Abstracts

**Published:** 1913-07

**Authors:** 


### SELECTIONS AND ABSTRACTS

### THE THERAPEUTIC CONQUEST OF SMALLPOX

seems to have been attained, according to the London news we publish in another column (page 319). It has long been known that the smallpox germ itself does not do much systematic damage, but the patient is really killed by the skin suppurations which occur after the system has succeeded in developing an immunity against the variola, and now it appears that if we give an artificial immunity against the pus organisms by means of vaccines, the patient is saved. All great discoveries are just as simple as this and yet we cannot help asking why did not some of our specialists think of it long ago, try it out and if successful, force everyone else to use it to end all deaths by smallpox even in those foolish people who refused to be immunized by Jennerian vaccine? Of course, these illogical folks have the same objection to anti-pus vaccines that they have to the Jennerian and might refuse to have the former administered if they do get smallpox, though it is remarkable how drowning men grasp at straws they flouted before they slipped into danger. So it is not at all sure that we can stop all smallpox deaths. At any rate, if the news given by Sir A. E. Wright is confirmed, it is one of the greatest therapeutic advances in the history of medicine. The poisons of the smallpox germ seem to reduce the skin's resistance to invasion by pus organisms, and a very respectable percentage of physicians believe that this effect is magnified by the injury which light rays invariably do to skins insufficiently protected by pigment. Not a few are convinced with the late Dr. Finsen that if we exclude the short rays of light soon enough the smallpox poison has not sufficient power of itself to destroy the anti-pus immunity we naturally possess. Surely all short rays are irritating to the skin and all other tissues they can reach, and more surely still, the lighter the country the worse are the pus infections following smallpox, and in the tropics the cases are dreadful. Denmark has so much cloudiness and so little light that it was possible to exclude the short rays from the patient and thus obtain excellent results in treating smallpox. The English in their dark land undoubtedly got some results from red curtains or that method would not have such a hold on the popular mind; but in very light countries such methods fail, probably because they do not exclude enough of the harmful short rays. The pus vaccines are then preeminently useful in lands of sunshine, but their effect in producing an immunity which cannot be beaten down by smallpox alone, would undoubtedly be lessened or perhaps nullified by exposure to excessive light. We therefore beg those who intend to try out the new treatment to be very careful to exclude sunlight from the start. The room lights should be greatly subdued and contain no short rays whatever. A red or yellow light is the only one permissible, preferably the red as it has the least actinic effect though it might be more irritating to the retina. This is particularly important in the tropics where the light can surely be blamed for much of the severity of smallpox in those of light complexion.

THE PRODUCTION OF JENNERIAN VACCINE FREE OF PUS GERMS is now a possibility. Hitherto we have been as clean as possible and depended upon glycerine or some other germicide to kill the organisms we could not exclude. Unfortunately these germicides will in time kill the vaccinia organisms also and though it is possible to prevent their multiplication by cold storage, it is difficult to transport them without warming them up. It is evident then, that a vaccine pus absolutely free of pus organisms is of vast advantage, for it will retain its vitality much longer and reduce the number of failures due to death of the vaccine. The new treatment, if proved to be even more successful than the first reports indicate, will not obviate the necessity for vaccination for a long time if ever—or at least until we learn how to avoid infection. The real nature of a case also may not be recognized until too late to do much good by pus-vaccines, though theoretically they should be successful at any stage. It may not be curable invariably, and therefore an artificial immunity is essential, and an absolutely safe vaccine must be used. If it is shown that calves first immunized to pus organisms do produce a far safer Jennerian vaccine, the proceeding should be made obligatory—not by law but by purchasers, particularly health authorities who buy and distribute vaccines. There are very rare instances of accidents in Jennerian vaccination and probably these will always occur no matter how careful we are, so it would be desirable to obviate the necessity of a preliminary immunization against pus. There is no doubt that in the far distant future we will eliminate smallpox from the face of the earth, and that it will never arise DE NOVO again from some non-pathogenic organism, but the time will not arrive until a generation or two after we have such complete control of the tropics, where the disease is now propagated, that we can vaccinate every child shortly after it is born. Then we can keep infection from the anti-vaccinationists who are largely responsible for the continued existence of the disease in civilized countries. We have long given up hope of attaining universal vaccination at home and this new possibility of curing smallpox makes us more hopeless still, as there are thousands who will decline to run the negligible risks of vaccination and prefer the greater risk of being cured in the rare chance of contracting smallpox. So we must turn our attention first to securing as complete a world immunity as possible. To do this we will need more vaccine than ever, and the necessity for a lymph free of pus germs is of increasing importance.

SPURIOUS VACCINATIONS must now be studied more carefully and we beg indulgence for again reverting to this hoary subject. The news that smallpox uncomplicated by pus infection is really a mild disease, directs attention to the fact that a proper Jennerian vaccination is also an exceedingly mild infection devoid of signs of active inflammation. We must repeat over and over again that laymen and not a few physicians erroneously consider the coccus inflammation as part of the process and that the worse the inflammation the better the protection. There is plenty of evidence that the facts are the opposite, and that vaccinia may be interfered with or even suppressed by staphylococcic and streptococcic invasion. This fully accounts for the number of cases of smallpox in vaccinated persons presenting hard, thick cicatrices indicative of tissue destruction. It also accounts for the large number of cases of alleged "successful" re-vaccinations in persons already protected by a proper infantile vaccination. As a dead lymph may give a skin reaction, it is also evident that many of these cases are incorrectly reported as successful re-vaccinations. As a matter of fact a successful re-vaccination is exceedingly difficult to produce in persons who have been properly protected, and it is not nearly so common as the profession generally believes. We must put an end to this false idea at once, for we hear that a scoundrel of a druggist has been in the habit of adding croton oil to his vaccine with the deliberate purpose of playing upon our error. His vaccine always caused a skin reaction and gained a great reputation for excellent quality and being always successful, though it may have been as inert as sawdust. The users were lulled into a sense of security though they were wholly unprotected. There is no question that cases of smallpox occurring in unprotected persons, who believed that a sore or inflamed arm was a vaccination, have given rise to much of the opposition to this operation. We ourselves were largely to blame, and the worst culprits among us were those medical schools which persistently neglected to teach the students what a successful vaccination really was. Comparatively few graduates from some colleges—a few years ago at least—had the slightest idea of vaccinia as a definite self-limited disease. We hope for a reformation without the necessity of calling in a layman to tell us how bad we really are. In the light of the new facts we must go over the whole subject anew to see if we have been harboring any other errors. For instance, we have been teaching that if a person is partly immune, a potent vaccine will begin to "take" but will be aborted later by the antibodies still present. These have been called successful vaccinations, though they may have been nothing more than the skin reactions of dead vaccines on persons already immune, or perhaps the action of croton oil. We have a lot to learn yet, so let us get at it. As for Sir Almoth E. Wright, the world will rise up and call him blessed.—EDITORIAL, AMERICAN MEDICINE.

### THE TREATMENT OF NEPHRITIS AND ALLIED CONDITIONS.*

MARTIN H. FISCHER, M. D., CINCINNATI.

(Continued From Last Issue.)

As I have already mentioned, any of the various apparatus on the market for intravenous injection may be used to give the alkaline hypertonic sodium chlorid solution intravenously. The

Fig. 6.—Pressure-bottle apparatus for injecting solution into vein.

medical attendant should choose the apparatus with which he is most familiar.

Perhaps that shown in Figure 2, with a cannula replacing the catheter and minus the thermometer and the insert F, is the simplest available form. The side tubulation with the rounded bottom or a glass bulb insert filled with glass wool (Fig. 3) will make it almost impossible, if care is used, to inject any sediment that may accidentally appear in the injection-fluid. The rate of inflow must be slow, and is conveniently regulated by gravity. The pressure-bottle arrangement shown in Figure 6 possesses some advantages over the apparatus just referred to. No special comment is necessary regarding its use, and I need not in this day emphasize the necessity of having all rubber tubes, etc., perfectly sterilized by boiling in distilled water.

The faults of the apparatus shown in Figure 6 are that it possesses no arrangement for keeping the solution at body-temperature, and that we do not know in as accurate a manner as we desire the exact rate and exact pressure at which the sodium chlorid-sodium carbonate solution is entering our patient at any moment.

To meet these difficulties, the very useful apparatus shown in Figure 7 was devised by Edmund M. Baehr. We have here again the same glass pressure-bottle already shown in Figure 6, but this is surrounded by a copper water-jacket by means of

Fig. 7.—Apparatus for keeping solution at body-temperature.

which the injection-fluid may be kept at body-temperature or a little above it. The thermometer registers the temperature existing in the packet, and as the temperature of the injection-fluid falls on its way into the patient, it is advantageously kept from 3 to 5 degrees above the temperature at which we wish to deliver the solution into the patient. The rubber bulb shown in Figure 6 is advantageously replaced in the apparatus shown in Figure 7 by a metallic pump; a mercury manometer, inserted as indicated in the drawing, allows one to know at all times the exact pressure obtaining in the pressure-bottle.

The solution should always be injected slowly into the circulation so that it may mix with the blood, and at as even a rate as possible. Not over from 30 to 40 c. c. should be injected per minute. By testing out the apparatus before making the injection it is easy to note just how much pressure is necessary to accomplish this. As the pressure in the larger veins is almost nil, from 30 to 40 mm. of mercury-pressure usually suffice, and one need never run above 50 mm. if a cannula or needle of proper diameter is chosen. If the solution is given to a patient who for any reason has to maintain an upright position, it is well to remember that in such a case the arm must be comfortably supported in as horizontal a position as possible in order not to have to work against a considerable hydrostatic pressure in the veins. The pressure and the oscillations of the mercury-column indicate every moment whether the solution is flowing properly or not.

The Quantity and Time-Interval of the Salt-Alkali Injections.—It is necessary to say now how much of the solution may be injected at one time and how often the injection may be repeated. In any case of suppression or in a case with convulsions, persistent vomiting or other alarming symptoms, from 1,800 to 2,000 c. c. of the solution should be given for the first dose. In the case of a child I give a proportionate dose obtained by dividing the child's weight by that of a small adult. A child of 30 kg. (66 pounds) is given half the dose of a man weighing 60 kg., etc. The repetition of the injection and the amount given subsequently must then be determined by the condition of the patient. If within two or three hours urine begins to come and the convulsions stop, or if the sensorium clears or the headache and eye-symptoms improve, enough has been given for the time being.

If the patient is awake, he is likely to complain of thirst during the injection. It is best not to let him satisfy his thirst immediately for the reasons that I have already discussed. If the redevelopment of some prominent symptom indicates that the patient is relapsing into his previous state, then a second injection of 1 or even 2 liters of solution may be given six, twelve, or twenty-four hours after the first injection. Closer rules than this can hardly be given. If the patient improves for a number of hours after the first injection, and then goes down again, repeat the injection at this time. From 500 to 1,500 c. c. in any twenty-four hours after the first injection is certainly safe.

If the suppression of urine is not absolute, then it is a useful guide as to the amount of alkali and salt that may be urged on the patient. It is safe to give alkali, and it should be given until the urine is PERSISTENTLY neutral or slightly alkaline to litmus. For self-evident reasons, it is possible for the urine to be alkaline immediately after an intravenous injection, even when the acid content of the body generally is still abnormally high. Not until the urine is persistently neutral, therefore, can we say that we have really succeeded in getting an adequate amount of alkali into the patient.

Various observers have commented on the large quantity of fluid that is injected intravenously in clinical cases of nephritis and allied conditions. I have injected as high as 4 liters (4 quarts) in twenty-four hours. Some clinicians have for various reasons remonstrated that this is dangerous, chiefly on mechanical grounds, and because this "throws work on the heart." Insofar as the elimination of water from the body costs energy, this is to a limited extent true, but only to a very limited extent, as I have previously insisted. Physiologists know that the volume of circulating blood can be more than doubled without appreciable effect. Counting the blood in the human being as one-thirteenth the body-weight, it is therefore entirely safe to inject a liter of fluid for every 13 kg. (about 28½ pounds)

of body-weight; but as I have pointed out elsewhere, (7), this is a safe figure if blood, in other words, water in combination with a colloid, is injected. Only such remains in the blood-vessels. When the water is injected "free," as in a salt solution, this rapidly leaves the blood-vessels, and so the amount of this that may be safely injected lies still higher. What I have said here is still largely true even when we deal with sclerosed blood-vessels, though to allow for the diminished elasticity of the blood-vessels in such cases a slower injection or injection of a less amount at more frequent intervals, as the judgment of the operator may dictate, may advantageously replace the single large injections.

About one in every three patients injected with alkaline hypertonic salt solution develops some reaction. At times he develops a chill which, however, does not last long, or he has a slight rise in temperature. Once I found sugar in the urine. It will be noticed that these findings are similar to those observed after intravenous injections of salvarsan and other medicaments. European authors have laid stress on the distilled water employed in making up the solutions, maintaining that bacterial products are present in old distilled water. For this reason, I have always urged the use of freshly distilled water, but even then I have seen these reactions. I next attributed the effect to the action of the alkali on the red-blood corpuscles, and a resulting hemolysis. This, however, can only be a small part of it, for one of the worst reactions I ever saw occurred in a woman to whom I gave a concentrated solution of neutral salt only. To explain the findings—which are now being studied on laboratory animals—I have come to the tentative conclusion that two things are active: first, a shrinking of the red blood-corpuscles which makes these less able to carry oxygen; second, a more important direct action of the salt and alkali on the medulla. The latter effect, I think, leads to the vasomotor disturbance in the skin which we call chill, to the

7. Fischer, M. H.: Edema, New York, 1910, p. 184; Nephritis, New York, 1912, p. 193; Kolloid-chem. Beihefte, 1911, ii, 304 (see Note 1), Hogan and Fischer, Kolloid-chem. Beihefte, 1912, iii, 385.

consequent retention of heat that accounts for the rise in temperature, and to the appearance of sugar in the urine, as Bock and Hoffmann, (8), found many years ago when rabbits were perfused with sodium chlorid solutions. How to avoid these effects is not yet entirely clear to me. The rules that I have formulated for my own guidance call for the use of freshly distilled water and as slow an injection as is conveniently possible. It is undoubtedly better to give in place of single large intravenous injections several smaller ones separated by intervals of time which allow the salt and alkali to diffuse into the body-tissues, but unfortunately the necessity of opening into more than one vein and the critical condition which we are usually asked to meet does not always allow of this. Before, throughout and after the injection the patient is carefully protected from muscular exertion, and the possibility of a chill is guarded against by extra blankets, hot-water bags, etc. If complete muscular and mental relaxation is not easily obtained, a SMALL dose of codein, heroin or morphine may be used.

### Clinical Results and Comment.

In the light of the considerations in the earlier paragraphs of this paper it will not surprise any one that the acuter forms of nephritis which we observe after various intoxications are usually easily relieved by giving alkali and salt in sufficient concentration. While at first sight it seems rather strange that a woman in a pregnancy-intoxication with little or no urine, with great quantities of albumin and casts, with constant headache and nausea, with partial blindness, with convulsions and coma perhaps, ceases her convulsions, comes out of her coma, says that her headache and nausea are better and shows an improvement in the urinary output within a few hours after an intravenous injection of an alkaline hypertonic salt solution, there is really nothing strange about it. If I am corerct in maintaining that these various signs and symptoms represent

8. Bock and Hoffman: Arch. f. (Anat. u.) Physiol. 1871. Fischer, M. H.: Univ. of California Publications in Physiology, 1903, i, 77: ibid., 1904, i, 87: Arch. f. d. ges. Physiol. (Pfluger's), 1904, cvi, 80: ibid., 1905, cix, 1.

from the side of various organs the same series of colloidal changes which in the kidney are called nephritis, and if I have correctly analyzed these colloidal changes as being of the nature that are induced through an abnormal production or accumulation of acid in these various organs (in its turn brought about by the same poison circulating through all the organs of the body), then it is to be expected that the alkali and salt which will relieve the signs and symptoms of these organs must relieve those referable to any other.

It is obvious that on such a basis an anesthesia-nephritis must be easily relieved when alkali and salt are introduced into the body. During an anesthesia we introduce into the body a poison which interferes with the normal oxidation chemistry in nearly all the cells; and that we have an abnormal production and accumulation of acid in the body following this is attested to not only by the thirst of which the patient complains, but by the accelerated breathing and heart-beat, the abnormally high acid (hydrogen ion) content of post-anesthetic urine, and the appearance in it of such "acidosis" products as acetone, diacetic acid, lactic acid, etc. As soon as we stop administering the anesthetic the patient begins to exhale this; therefore, in a comparatively short time the intoxication responsible for the abnormal production and accumulation of acid in the body disappears.

The patient also usually succeeds in oxidizing the acid products resulting from his intoxication, so that it is the usual thing to see a patient bear his anesthesia without bad after-effects. Sometimes, however, he is not so successful in overcoming the effects of his anesthesia, and then if his intoxication evidences itself chiefly by symptoms referable to his kidneys, we say he has a post-operative nephritis. If it should happen to involve his liver particularly, we say he has a "postoperative jaundice," "chloroform liver," etc. If now we administer alkali and salt, the alkali neutralizes the abnormal acids present, and the increased salt concentration reduces the hydration-capacity of his swollen kidney-colloids and other body-colloids. If the injury to them has not been too great (or technically, if the colloidal changes have not become "irreversible"), these measures quickly restore his kidneys (and other involved organs) to a more normal state. As the body-colloids shrink, "free" water becomes available for urine. This shrinking in the kidney assures this organ a better blood-supply and the kidney-cells themselves are also once more enabled to resume their work. Once we have obtained this result, a recurrence need scarcely be feared, for the original toxin (the anesthetic) has by this time disappeared from the body and the relief obtained is permanent.

If we write the names of other toxic agents in the place of "anesthetic," we can imagine what happens in the nephritides that we encounter in any of the acute infections, and in the various other acute intoxications which we know to be associated with the development of nephritis. Here again natural physiologic processes take care of the great majority of cases, but when they do not we may again be able to give relief.

For self-evident reasons our prognosis grows worse and our efforts need to be greater when the intoxication is prolonged or is of a less removable type. We encounter this situation in protracted infections, in acute infections that leave behind toxins that stick with particular firmness to the kidney-cells (scarlet fever?) and in the prolonged intoxications resulting from phosphorus and the heavy metals. Not alone do some of these produce irreversible colloidal changes (necrosis) in the cells of the kidney from the start—changes, therefore, which can never be "cured" by any therapeutic procedure—but, even when such is not the case, in these prolonged intoxications the interference with the normal oxidation chemistry of the kidney-cells is of a more lasting character. Here our therapy also must be more persistent. Therefore in the nephritides following the administration of mercury or arsenic, in those accompanying acute and chronic infections, and in those of pregnant women, a single dose of alkali and salt may do no more than give temporary relief. We must meet the intoxication as long as it persists, and only as the nature of the intoxication permits of this, or our skill is equal to the occasion, can be hope for good results.

We move from the protracted case of nephritis which is the result of a lasting intoxication of some kind by imperceptible steps over into the chronic nephritides. As soon as we discuss the chronic nephritides we find that we have to distinguish between those which represent a mere continuation of what was once a more acute process (the chronic parenchymatous nephritides, the secondarily contracted kidneys) and those which are chronic from the start, as in the type generally known as chronic interstitial nephritis (primarily contracted kidney) associated with changes in the cardiovascular system. Evidently if my views are correct, the nephritis which continues because of a protracted intoxication needs equally protracted treatment with alkali, salt and water. It has seemed to me that such a procedure yields good, and at times unexpectedly good results; but of this each must convince himself, for it is impossible, except as one works these things out for himself, adequately to meet the eternal argument that what has happened in such cases would have happened anyway.

Especially is it difficult to meet such an argument when we deal with the chronic interstitial types of nephritis associated with cardiovascular disease. One can foresee from the start that this type of nephritis offers the least possible chance of being markedly benefited by an alkali-salt-water therapy, and in its final stage none at all. I have emphasized this repeatedly, and were it not for the fact that it is on this very type of case that some of my critics have based their arguments, it would scarcely be necessary to refer again to some self-evident facts. How much and what can we do for such cases?

The primary change in chronic interstitial nephritis associated with vascular disease is not nephritis but vascular disease. Every experimental fact and all physiologic reasoning bear this out. (9.) In consequence of the vascular disease, one piece after another of the kidney suffers destruction. These pieces as they degenerate show the morphologic evidences of "parenchymatous nephritis," and corresponding with these destructive

---

9. For a discussion, see Fischer, M. H.: Edema, New York, 1910, p. 1912. Nephritis, New York, 1912, pp. 56 and 94.

lesions we find traces of albumin and a few casts in the urine. This process may go on for years without being noticed by the patient, for the healthy kidney-tissue between the areas of nephritis is able to carry on the necessary functions of excretion. Such a patient can get along easily with one-fourth of his total kidney-substance, and is more likely first to be made aware of his condition in a life-insurance office than in that of a physician whom he has himself sought; and if we make the diagnosis, his blood-pressure and the evident changes in his heart and blood-vessels are what lead to a correct estimate of the significance of his albumin and casts.

If we but remember that the blood-vessel disease is the primary thing, then we get also a correct estimate of the significance of the changes in distant organs—changes considered by most as consequences of chronic interstitial nephritis. An "albuminuric retinitis," a glaucoma, persistent headaches, even the uremic convulsions and coma occurring in these cases, are the same thing in the retina, the eye or the brain which in the kidney we call chronic nephritis. The hemorrhage into and swelling of the retina, the swelling of the eye with its clouding of the clear media, and the edema of the brain are analogous to the hemorrhage, the clouding and the swelling of smaller or larger areas of the kidney affected with chronic interstitial nephritis. That which is common to both is disease of the blood-vessels; and as this blood-vessel disease cannot be and is not materially influenced by the injection of alkali, salt and water, this therapeutic procedure can be of little or no use in this type of disease. The only cases in which it can be of service are those in which the blood-vessel disease is in itself not wholly responsible for the observed changes, but other temporarily active factors have been or are also responsible in bringing about the clinical picture.

An illustration of this is offered by the "exacerbations" which we note clinically in the cardiovascular types of chronic interstitial nephritis. Cardiac dilatation, an anesthetic, an alcoholic debauch, hard muscular work or starvation will do to the cardiovascular kidney what they do to a normal kidney, in which case these effects are added to those already produced in this organ by the irremovable blood-vessel disease. The cardiovascular (focal) parenchymatous nephritis becomes a diffuse nephritis, and clinically, a normal water output with few casts and little albumin gives way to a lowered output with many casts and much albumin. Alkali and salt relieve the consequences of such added factors, but blood-vessel changes that permanently interfere with the blood-supply (especially end-arteries) to a portion or all of an organ, are not relieved thus.

If the simple argument that I have outlined here will be borne in mind, it will help to a better understanding of what may be expected and what cannot be expected from the use of alkali, salt and water in the chronic types of nephritis. The final picture of such cases is all too often not that of pure nephritis, but one complicated by a failing heart. When a heart from any cause whatsoever drops below the lowest level of efficiency necessary to maintain a proper circulation, and has no remnants of recuperative powers left in it, alkali and salt cannot supply them. (10.)

A detailed account of the clinical histories of a number of nephritics treated by the method herein described is given

---

10. This is the type of case chosen by Joseph L. Miller (Am. Jour. Med. Sc., 1912, clxiv, 8; Clinical Experience in the Drug Treatment of Edema, abstr. The Journal A. M. A., June 22, 1912, p. 1972), on which to test out the value of a salt-alkali therapy. Aside from the fact that his "'Fischer' solution" had a composition all his own, his results were entirely predictable. According to his own statement the majority of his patients were nephritics with permanently decompensated hearts. Naturally alkali and salt could not produce a diuresis when the mechanism for water secretion was about gone. Only heart and respiratory stimulants, such as caffein and its derivatives, drugs, in other words, which gave these hearts a last kick and temporarily assured a better oxygen supply to the kidney- and body-tissues generally, gave a temporary "diuresis." It was not necessary to be a believer in any of the colloid notions of water absorption to foresee all this, for we have known since 1860 that an inadequate circulation will not allow even a normal kidney to secrete urine.

not only in my book but elsewhere. (11.) These have been commented on in various ways—to some they "read like the cures of the nostrum venders," (12) to others the patients would have recovered anyway; some believe the whole procedure valueless, some believe it distinctly harmful; one group accepts what I have written as essentially true. In the dilemma I can only advise the objective thinker in medicine to reject my views and in treating his patient first exhaust other approved methods. If by any chance he should feel that his patient is going to die, then he may turn to an alkaline hypertonic salt solution. If the patient dies, the expected will merely have happened. If the patient lives, it proves nothing, but it may encourage repetition of the experiment. And this, I feel, is all that is necessary.—Jour. A. M. A., May 31, 1913.

11. Fischer, M. H.: Nephritis, New York, 1912, p. 160; Tr. Assn., Am. Phys., 1912.

12. Review of Fischer's "Nephritis," Arch. Int. Med., May, 1912, p. 637.

### REINOCULATION WITH FRIEDMANN'S VACCINE.

On May 29, 1913, the Board of Health, with the approval of the following members of its Medical Advisory Board, Dr. Joseph E. Bryant, Chairman; Dr. T. Mitchell Prudden, Secretary; Dr. Abraham Jacobi, Dr. Simon Flexner, Dr. A. Alexander Smith, Dr. John Winters Brannan, Dr. L. Emmett Holt, and Dr. Walter B. James, adopted a resolution providing for the official supervision of immunization with living bacteria, and stating that "the use of living bacterial organisms in the inoculation of human beings for the prevention or treatment of disease shall be and is hereby prohibited in New York City until after full and complete data regarding the method of use, including a specimen of culture and other agents employed therewith and a full account of the details of preparation, dosage and administration shall have been granted in writing to the Board for the use of the same." It will be remembered that a short time before, a company which had purchased the rights of Friedmann's vaccine had opened an institution in this City and proposed to establish others throughout the country for the treatment of tuberculosis by Friedmann's methd. The effect of the action taken by the Board of Health was to close the Friedmann Institute in New York City, pending a decision on the application for a permit under the new rule. As a number of persons had already been treated at various hospitals and at the Friedmann Institute, permission was requested by the Institute to continue treatment in these cases.

On June 17 the Board of Health adopted a resolution which forbids the use of the treatment, except in respect of cases already treated, and prescribes very minutely the conditions under which reinjections may be made. This resolution and the decisions later reached thereunder have the effect of denying the applications of the Friedmann Institute. The resolution reads as follows:

1. The injection or treatment of human beings within The City of New York with the F. F. Friedmann "vaccine" (which contains living bacterial organisms), is prohibited, except under the following regulations and restrictions:

2. No person may be treated, according to the foregoing, who did not receive one or more such treatments with the same kind of living bacterial organisms, in New York City, previous to May 30, 1913.

3. These treatments may be administered only in the following named hospitals: Bellevue Hospital, Mt. Sinai Hospital, Montefiore Home and Seton Hospital, and any other that the Board of Health may approve when arrangements have been made for proper observation and control of the cases.

4. Only those persons may be so treated who reside in one of the hospitals named above or who agree to return to the hospital in which they receive such treatment as often and at such times as the physicians in charge of said hospital deem it advisable for their proper observation.

5. No person may receive treatment according to the foregoing until he or she has presented to the physician administering the treatment a fac simile of the following form, filled out and signed, in duplicate, by a physician who is duly authorized to practice medicine in New York City and who is not in any way financially interested in the treatment:

I hereby certify that I have examined M . . . . . . . . . . . . . . . Age, . . . . . . . . . . . . . . . . . . . . Sex, . . . . . . . . . . . . . . . . residing at . . . . . . . . . . . . . . . . . . . . . . . . . . . . . . . . . , that received treatment with . . . . . . . . . . . . . . , in New York City on the following dates: . . . . . . . . . . . . . . . . . . . . . . . . and I recommend that . . . . . . be permitted to receive additional treatments with same kind of living bacterial organisms under the regulations prescribed by the Board of Health of New York City.

Date, . . . . . . . . . . . . . . Signed, . . . . . . . . . . . , M. D.,

Address . . . . . . . . . . . .

No person may be so treated until he or his parent or guardian, in case of a minor, has signed a declaration, in duplicate, stating that he desires these treatments continued.

One copy of each of the above declarations (certificate by physician and declaration by patient, parent or guardian) must be filed in the Department of Health on or before the day such treatment is administered, and the other copies retained by the physician who administers the treatment. This physicaan must also file in the Department of Health a statement of the dose given and the method of its administration, and the hospital in which it was given, in each case within 48 hours after treatment.—Department of Health of New York City.

---

### HORMONES.

On the firing line of medical progress we find the April number of The Prescriber, Edinburgh, devoted to the subject of Hormones. We present below some interesting selections.

### Introduction

Yesterday was the day of the pathologist, more especially of the bacteriologist. The day before yesterday witnessed the triumphal progress of Surgery. Physiology and Medicine, all wrung in the withers and quite chapfallen, contented themselves, perforce, with the crumbs that fell from the rich microbic tables of the others. But the whirligig of time brings in his revenges. To-day and to-morrow, and the day after, are foreordained to the physiologist, the physician, and the therapist. Their hour has come through the agency of the internal secretory glands, which already unfold before the astonished view of the seeing eye, a land of promise beside which the vast territories conquered by Lister and Pasteur are destined to pale into honorable insignificance. The ductless glands and their hormones come to us as peaceful conquerors who brook no denial. They lighten our darknesses and show us miracles. In studying them and endeavoring to unravel their intricate and esoteric mysteries, one seems ever and anon to be on the trail of the Great Secret, and in danger of losing one's mental perspective. The Editor of The Prescriber, in collecting and collating so much that is of real and enduring value in one issue of the journal, has rendered a great service to the large and daily increasing number of those who hearken to the call of these seductive voices.

LEONARD WILLIAMS.

### HORMONE THERAPY—PAST AND PRESENT.

Like many other modern "discoveries," hormone therapy had its prototype in antiquity. The use of animal organs in the treatment of disease is as old as medicine itself. The ancient Egyptians treated various diseases by means of animal secretions, and in the Ayurvedic writings of Sushruta, who lived some time between 400 and 600 B. C., mention is made of the use of orchitic substance in the treatment of impotence. This is probably the earliest specific reference to organotherapy, and since that time it has never been absent. The works of Pliny and Galen contain numerous references to the employment of animal organs in the treatment of diseases of the corresponding organs in man. The principle of isopathy, or SIMILIA SIMILIBUS CURANTUR, led to the establishment of the popular, and certainly harmless, system of homeopathy, but the more repulsive forms of animal therapeutics long held sway. At the present day we have such animal products as cod-liver oil enjoying the full benefit of official recognition.

Of course, all these remedies were empirical. Indeed there was even a tinge of superstition in many of the cures,—that superstition which allows the ignorant to attain by intuition what the learned can arrive at only after years of toil and study. But some twenty years ago a new phase of so-called rational therapeutics sprang up—a system wherein gland substances were administered in cases where disease was believed to be due to deficient secretion of that particular organ. What the gland secreted, and how the deficiency affected the disease, was then but imperfectly known; still the adrenals and the thyroid were gradually having a responsibility placed upon them, and these glands, obtained from animals, were used empirically in almost every conceivable condition.

Then followed what might be called a craze for organotherapy. Every internal organ of the sheep or ox, from the brain to the testes, was removed and served with pharmaceutical skill in the form of dessicated powder, compressed tablets, or glycerin extract. Doubtless benefit was derived from the treatment, but soon much of it was discarded, although in France OPOTHERAPIE is to-day as popular as ever it was. Then search was made for active principles in these organs, and in 1901 Takamine and Aldrich independently discovered adrenaline, the active principle of the adrenals. Since then great strides have been made, but it has been left to Starling and his co-workers to determine the actual relations of the internal secretions to physiology.

One of Starling's earliest pronouncements on the subject is to be found in a profoundly interesting and illuminating article on "The Chemical Co-ordination of the Activities of the Body," which appeared in Science Progress for April, 1907. In this article the author refers to the excitatory substances (Reizstoffe), long familiar to botanists, which have a profound dynamic effect on the living cell. In this respect they present a close analogy to the substances that form the ordinary drugs of our pharmacopoeias. We cannot do better here than quote Starling's own words:

"Since in the normal functioning of the body they have to be discharged at frequent intervals into the blood-streams, and carried onward by this to the organ on which they exercise their specific effect, they cannot belong to that class of complex bodies, which include the toxins, of animal or vegetable origin. . . . . We must therefore conceive them as substances, produced often in the normal metabolism of certain cells, of definite chemical composition, and comparable in their chemical nature and mode of action to drugs of specific action, such as the alkaloids. This conclusion is borne out by the few investigations which have been made as to the nature of the chemical messengers in the case of certain well-marked correlations of function in the higher animals. In consequence of the distinctive features of this class of bodies, and the important functions played by them in the higher organisms, I have proposed to give a special name to the class—viz., HORMONES, from ORMAW (I arouse or excite.")

This, then, is the beginning of hormone therapy, the explanation of that system of animal medication so steadfastly believed in throughout the ages. The body, it would appear, manufactures its own drugs. These are supplied by the glands of internal secretion; and not only have they the power to correlate and co-ordinate the various bodily functions—such as pregnancy and mammary secretion, growth and sexual development, and so forth—but they also destroy toxins, and further still, these hormones CONTROL ONE ANOTHER. This last is a most important phase of hormone therapy; for we must remember that when a certain secretion is deficient we not only have the results of this deficiency to deal with, but we have also the baneful effects of an uncontrolled antagonistic secretion. This "hormone balance" is a most important factor in medical treatment, and it is not too much to say that it is very frequently overlooked.

---

Bilateral inuinal hernia in female infants often contain the Fallopian tubes and the ovaries.—AMERICAN JOURNAL OF SURGERY.

## Figures and Tables

**Fig. 6. f1:**
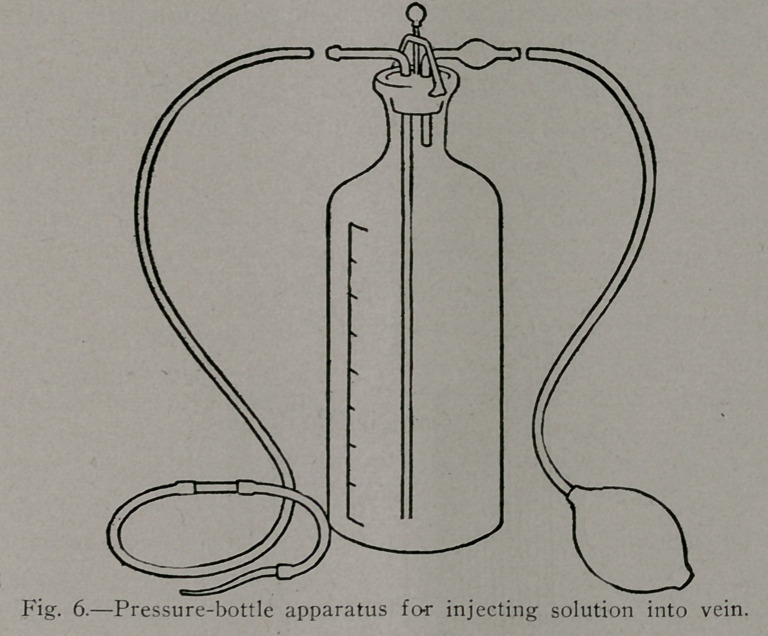


**Fig. 7. f2:**